# Pharmacokinetics and pharmacodynamics of febuxostat under fasting conditions in healthy individuals

**DOI:** 10.3892/etm.2013.1414

**Published:** 2013-11-19

**Authors:** MEI ZHANG, XIAOHUI DI, LIN XU, JUAN XU, YONGGE YANG, NAN JIANG, LIXUE SONG, XUETING XU

**Affiliations:** Department of Pharmacology, General Hospital of Beijing Military Command, Beijing 100700, P.R. China

**Keywords:** febuxostat, pharmacokinetics, pharmacodynamics, serum uric acid

## Abstract

The aim of the present study was to investigate the pharmacokinetic and pharmacodynamic characteristics of febuxostat following the administration of single and multiple oral doses under fasting conditions to healthy individuals. Thirty-six healthy subjects were randomly divided into three groups, each containing 12 subjects (six male and six female) as follows: Group A, treated with a single oral dose of febuxostat (40 mg); group B, treated with a single oral dose of febuxostat (80 mg) followed by multiple oral doses of febuxostat for 7 days; and group C, treated with a single oral dose of febuxostat (120 mg). Blood samples were collected, and the plasma drug levels and serum uric acid (UA) concentrations were determined by clinical laboratory testing. Febuxostat displayed a linear pharmacokinetic profile for oral doses of 40 to 120 mg. Drug accumulation was not detected following multiple oral doses. When febuxostat was administered as single doses of 40, 80 and 120 mg, the 24-h UA concentration (UA24) values displayed a linear correlation with the dosage. The relationship between UA24 and the three single dose levels (40, 80 and 120 mg) was analyzed. The difference in UA24 between every single dose was significant (P<0.05). After 3 and 7 days of dosing, reductions of 46.67 and 52.69%, respectively, were observed in UA24. On day 7 of dosing, the mean reduction in the UA concentration was 51.83±7.00%. This study demonstrates that febuxostat reduces serum UA concentrations in a dose-linear manner.

## Introduction

Hyperuricemia, characterized by high serum levels of uric acid (UA), is frequently reported in mainland China (1). Several factors, including genetic background, drug metabolism, kidney disease, and a high dietary intake of purines and proteins, are associated with the pathogenesis of hyperuricemia. These factors may induce aberrant metabolism of purines resulting in excessive UA formation (2,3). According to the literature, in ~10% of cases of hyperuricemia, UA accumulates in the form of a sodium salt that is deposited in the joints, soft tissues, cartilage and kidney, which may promote inflammatory reactions and eventually lead to gout (4).

Febuxostat, a urate-lowering drug, has been indicated for the treatment of hyperuricemia and chronic gout (5,6). The drug acts a xanthine oxidase inhibitor and is able to induce reductions in serum UA levels (7). In the present study, single and multiple doses of febuxostat were administered to healthy Chinese subjects. The aim of the study was to investigate the pharmacokinetic and pharmacodynamic parameters of febuxostat. In addition, the concentration-time curve for febuxostat was determined and analyzed, in order to obtain information that may be helpful in the clinical management of patients with hyperuricemia and gout.

## Subjects and methods

### Subjects

A total of 36 healthy subjects (male, 18; female, 18; aged 19–40 years old; BMI, 18–24) were enrolled in the study. Key inclusion criteria were: normal ECG, negative urine pregnancy test resukts, and values within the normal range for standard laboratory tests of blood, urine and biochemical parameters. Exclusion criteria included treatment with traditional Chinese or Western medicine within 2 weeks prior to the investigation, and blood donation or clinical tests for other medicines within the previous 3 months. All subjects provided informed consent. This study was approved by the Ethics Committee of the General Hospital of Beijing Military Command (Beijing, China). The subjects were randomized into three febuxostat dose groups, each containing 12 subjects (six male and six female) as follows: Group A, single oral administration (40 mg); group B, single oral administration (80 mg) followed by multiple oral doses for 7 days; and group C, single oral administration (120 mg). All subjects received oral febuxostat under fasting conditions. Subsequently, the subjects were observed to identify any adverse effects or complications.

### Treatment groups

In the single-dose group, 5 ml venous blood was collected from each subject prior to febuxostat administration and at 0.25, 0.5, 1, 1.5, 2, 3, 4, 6, 8, 10, 12, 16 and 24 h after febuxostat administration. In the multiple-dose group, 2 ml venous blood was collected from each subject at 0, 5, 10 and 24 h on the day prior to febuxostat administration (day -1). From day 1 to day 7, 5 ml venous blood was collected from each subject prior to febuxostat administration. In addition, on day 7, 5 ml venous blood was collected from each subject at 0.25, 0.5, 1, 1.5, 2, 3, 4, 6, 8, 10, 12, 16 and 24 h after the administration of febuxostat to determine drug level.

For each sample, 2 ml blood was used for assessment of the UA concentration. The remaining 3 ml of blood was centrifuged at 351 × g in a heparin tube, and the serum was stored at −80°C for drug concentration testing.

### Drug concentration testing

Drug concentration testing was performed according to previously described methods (8,9). In brief, 100 μl serum was mixed with the internal standard (bezafibrate 50 μg/ml, 10 μl, purchased from the National Institute for Drug and Food Control, Beijing, China). Acetonitrile (300 μl) was added to the sample, which was centrifuged at 250 × g for 2 min and then at 1,204 × g for 1 min. The supernatant (10 μl) was collected for analysis.

Plasma concentrations of febuxostat were determined using a validated high-performance liquid chromatography (HPLC) method (8,9). The serum samples did not contain any endogenous materials that interfered with the evaluation of febuxostat or the internal standard. Calibration curves for febuxostat were linear for concentrations ranging from 10 to 8,000 ng/ml (R^2^>0.995), which was consistent with the required analytic sensitivity. Appropriate precision and accuracy was achieved as the results of inter- and intra-laboratory comparisons in high, middle and low concentrates were accepted with a relative SD of <15% in the present study. The recovery rate of the extractions was >70%. According to guidelines for the research of chemical drug and clinical pharmacokinetics (3), after standing at room temperature for 4 h, the samples underwent three melt/freeze cycles at −80°C, were stored at −80°C for 24 days, and then placed in a sampler after sample treatment for 24 h.

### Analysis of UA concentration

UA is considered to be a pharmacodynamic marker of febuxostat as the therapeutic effect of febuxostat depends on reductions in UA concentrations. For each sample, 2 ml blood was examined using a Roche Modular P800 Automatic Biochemistry Analyzer (Roche Hong Kong Ltd., Hong Kong, China) to measure the UA concentration.

### Statistical analysis

Data are expressed as the mean ±SD. Noncompartmental pharmacokinetic analysis was performed using the Drug and Statistics software (DAS 2.0) as described previously (10). P<0.05 was considered to indicate a statistically significant difference.

Actual measured UA values were adopted for pharmacodynamic analysis. Quantitative descriptions and statistics of pharmacodynamic parameters are displayed using mean, SD, geometric mean, statistical charts and diagrams. The correlation between dose and pharmacodynamic parameters was evaluated using Microsoft Office Excel 2003.

## Results

### Pharmacokinetic parameters and concentration-time curve of febuxostat

**Figure 1:**
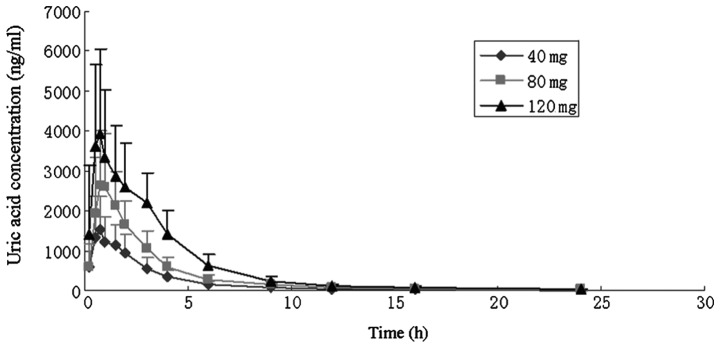
Plasma concentration-time curve of febuxostat after single oral doses of 40, 80 and 120 mg under fasting conditions in healthy subjects.

**Figure 2:**
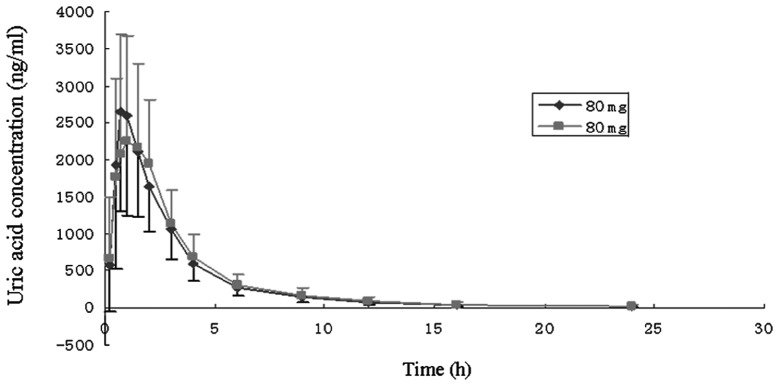
Plasma concentration-time curve of febuxostat after single and multiple oral doses under fasting conditions in healthy subjects.

With regard to pharmacokinetic parameters, no significant difference was noted between genders in the single-dose groups. In the multiple-dose group (80 mg, once-daily for 7 days), no statistical difference was identified in the drug absorption degree and rate. There was no evidence of drug accumulation. The main pharmacokinetic parameters are shown in Table I, and the mean concentration-time curves for each dose group are illustrated in Figs. 1 and 2.

### Pharmacodynamic parameters of febuxostat

**Figure 3:**
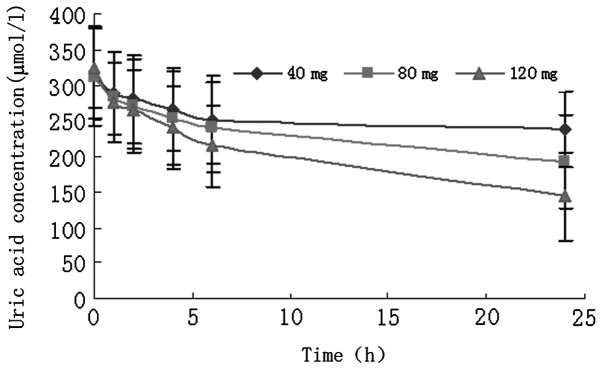
Mean effect (UA concentration)-time curve of febuxostat after single oral doses of 40, 80 and 120 mg under fasting conditions in healthy subjects. UA, uric acid.

**Figure 4:**
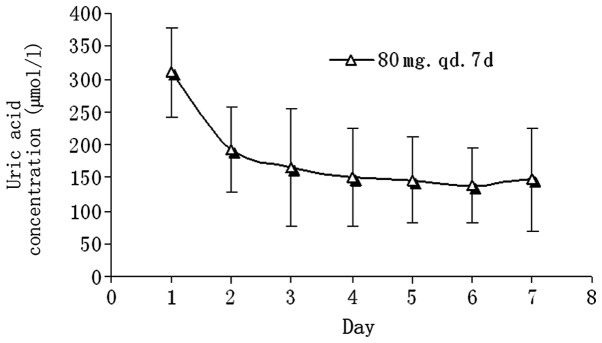
Mean effect (UA concentration)-time curve of febuxostat after multiple oral doses under fasting conditions in healthy subjects. UA, uric acid.

In all three single-dose groups, decreased UA activity was observed 1 h after febuxostat administration (Fig. 3). Regression analyses and Student’s t-test were used to determine the regression coefficient. The linear relationship between febuxostat dose and 24-h UA concentration (UA24) was expressed as follows: y = −1.16× + 283.95 (R^2^=0.9994). ANOVA evaluation of UA24/dose revealed significant differences between dose groups (P<0.05). In the multiple-dose group, compared with baseline, reductions in UA concentrations of 38.13, 46.67, 51.89, 52.93, 55.77 and 52.69% were noted on days 2, 3, 4, 5, 6 and 7, respectively. After 4 consecutive days of treatment with febuxostat, the UA levels in the subjects were low (Fig. 4).

### Comparison of UA concentrations prior to and following febuxostat administration

**Figure 5:**
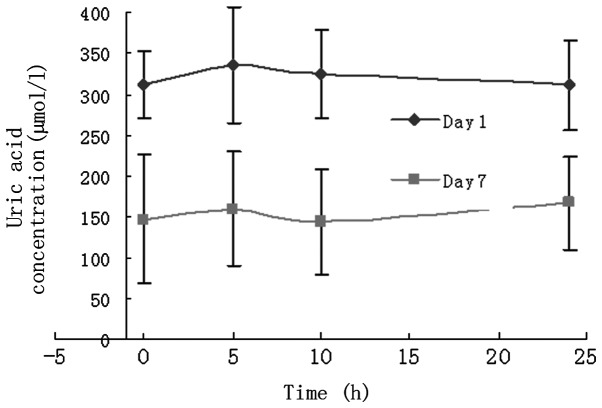
Pre-dose and 24-h mean UA concentration-time profiles following daily oral doses of febuxostat (80 mg) under fasting conditions in healthy subjects. UA, uric acid

On day -1 and 7 days after febuxostat administration, the concentration of UA was measured at 0, 5, 10 and 24 h, respectively. The reduction of the UA concentration on day 7 compared with that on day -1 (baseline) suggests that febuxostat has a therapeutic effect (Fig. 5). The mean reduction in the UA concentration on day 7 relative to that on day -1 was 51.83±7.00%.

### Safety evaluation

Among the 36 healthy subjects, leukocytes were detected in the urine of two male subjects who received a single 40-mg dose of febuxostat. In addition, one female subject in the multiple-dose group reported dizziness and drowsiness, which resolved spontaneously after a meal. These symptoms may have had no association with febuxostat usage. No drug-related side-effects and/or severe adverse events were observed in the subjects in this study.

For all 36 healthy subjects, serum UA concentrations were re-assessed 48 h after febuxostat was discontinued. The results indicated that the UA concentrations had returned to the normal range (mean, 301.56±13.34 μmol/l; normal, 119–416 μmol/l).

## Discussion

Febuxostat, a highly selective inhibitor of xanthine oxidase, is a novel drug for the management of hyperuricemia plus gout. The therapeutic effect of febuxostat mainly depends on its ability to lower UA concentrations. In the present study, febuxostat reduced UA24 in a dose-dependent manner in healthy subjects, which was consistent with a previous study (11). In the 40-, 80- and 120-mg single-dose groups, the mean reduction in UA24 was 25.38, 38.13 and 55.69%, respectively.

In the present study, linear absorption of febuxostat was observed across the dose range after single and multiple doses, which was supported by the fact that the increasing dose levels and administration of multiple doses had no effect on the T_max_ or the dose-normalised C_max_ of febuxostat. Moreover, our study was consistent with a previous study (11), which indicated that the volume of distribution of febuxostat was not affected by increases in dose level and the administration of multiple doses. Based on this finding, it may be speculated that the dose level and schedule of febuxostat administration did not affect tissue distribution *in vivo*.

According to UA testing, the renal clearance of febuxostat had no significant role in the elimination of the drug *in vivo* as only a small proportion of the drug remained unchanged. Under different drug concentrations, the increase of AUC was partly due to the increase of the concentration. This phenomenon was most likely due to the potential increase in renal clearance.

For the majority of drugs, pharmacodynamic effects appear later than would be predicted by plasma concentration levels because serum is not their site of action. In the present study, the serum UA concentration was considered to be a therapeutic marker mainly to facilitate sampling and testing. It was also confirmed that there was a time delay between peak plasma drug concentrations and the therapeutic effects of febuxostat.

Notably, gout attacks may become more frequent in patients with hyperuricemia plus gout when febuxostat is taken at an early stage (5). It has been postulated that this may be due to the mobilization of urate from tissue deposits as the serum UA concentration begins to fall. Therefore, it is to be emphasized that our conclusions are limited to healthy subjects.

In this study, febuxostat was orally administered to healthy subjects and changes in UA concentrations were measured, based on which the pharmacological activity of febuxostat was investigated. Our results demonstrate that single doses of febuxostat are associated with dose-dependent reductions in UA concentrations. After 3 days of continuous febuxostat administration, UA levels remained low (145.17±10.31 μmol/l). The mean reduction in the UA concentration on day 7 relative to that on day -1 was 51.83±7.00%. The concentrations of UA returned to normal 48 h after febuxostat was discontinued.

## Figures and Tables

**Table I:** Pharmacokinetic parameters of febuxostat after single and multiple oral doses under fasting conditions in healthy subjects

	Dose level			
	
Parameters	40 mg	80 mg	120 mg	80 mg/qd/7d
AUC_0–24h_ (ng·h/ml)	4536.6±1382.3	8216.0±2873.2	14404.1±4132.7	-
T_1/2_ (h)	3.81±1.77	5.02±1.23	3.91±1.52	-
T_max_ (h)	1.02±0.72	0.96±0.35	1.21±0.95	-
CL/F (l/h)	9.41±2.87	10.92±4.66	8.97±2.81	-
Vd/F (l)	48.80±19.49	77.90±32.35	49.01±21.63	-
C_max_ (ng/ml)	1911.95±678.40	2966.70±1176.13	4868.61±1792.14	-
C_max ss_ (ng/ml)	-	-	-	2957.07±1290.74
C_min ss_ (ng/ml)	-	-	-	25.39±12.52
C_av ss_ (ng/ml)	-	-	-	360.29±149.62
DF	-	-	-	8.23±2.11
Rac	-	-	-	1.14±0.54

AUC_0–24h_, Area under the plasma concentration-time curve; T_1/2_, half-life; T_max_, peak time; CL/F, clearance; Vd/F, apparent volume of distribution; C_max_, peak concentration; C_max ss_, maximum concentration at steady state; C_min ss_, minimum concentration at steady state; C_av ss_, average plasma concentration at steady state; DF, fluctuation coefficient; Rac, accumulation ratio.
